# Staged Cataract Post Scar Removal: A Case Report

**DOI:** 10.7759/cureus.9145

**Published:** 2020-07-11

**Authors:** Aaishwariya Gulani, Arun C Gulani

**Affiliations:** 1 Medicine, University of Central Florida, Orlando, USA; 2 Ophthalmology, Gulani Vision Institute, Jacksonville, USA

**Keywords:** cataract, keratoconus, cornea, corneal scar

## Abstract

A 62-year-old white Caucasian male was referred with Keratoconus and posterior subcapsular cataract (PSC) and a history of 12 previous procedures including implantable contact lens surgery (ICL), Intacs corneal implant, Cross Linking, Phototherapeutic Keratectomy (PTK) and Photorefractive Keratectomy (PRK). All the procedures were surgically well done by his surgeon but resulted in 20/200 vision and an irregular, scarred cornea. After undergoing Laser Corneoplastique and a staged Cataract surgery, he was brought to unaided 20/20 vision in this eye.

## Introduction

Corneal scar cases are often met with transplants as a solution. However, this technique is invasive and often presents with rejection within a year of the transplant. Thus, newer techniques are being used to replace a corneal transplant for scar patients. In this patient, Laser Corneoplastique was done followed by cataract surgery [1]. Staging his surgeries allowed one surgery’s results to provide a better base for the following surgery and bring this patient to emmetropia.

## Case presentation

A 62-year-old male was referred with a diagnosis of Keratoconus and past history of 12 procedures. He had a best-corrected vision of 20/200 and an irregular cornea with scars and a posterior subcapsular cataract (PSC).

The first step of his procedure was a preparatory Laser Corneoplastique procedure [1]. We refracted the patient’s scarred cornea and planned a surface Excimer modified Photorefractive Keratectomy (PRK) surgery to a clear and measurable endpoint. The Laser was refractively applied using +0.25, -0.75 x 010 to ablate a total of 4 microns with 39 pulses. This brought him to a post-op day one uncorrected vision of 20/70, best-corrected to 20/30. The patient was already pleased with his vision improvement to an extent that he opted to wait for nearly a year for the planned cataract surgery. We took advantage of this waiting interval to confirm the patient’s corneal measurability and stability to plan for his staged, cataract surgery.

**Figure 1:**
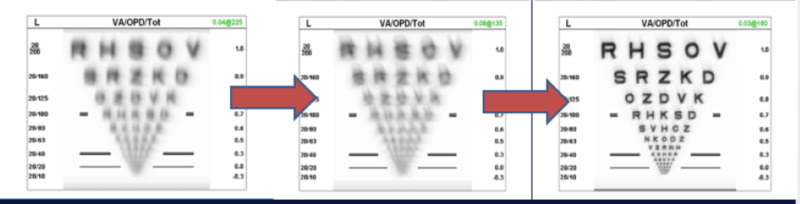
Vision improvement over staged surgery to 20/20 Patient went from 20/200 to 20/70 to 20/20 over the course of his staged procedure

Twelve months later, he came in duly for his cataract surgery after noticing diminishing vision. Though his cornea was now more measurable, he had had multiple corneal surgeries which could impact accuracy, thus, we decided to leave his Intacs corneal implants in place and stage his Cataract procedure to first remove his implantable corneal lens (ICL) and Cataract to leave him aphakic to then avail of accurate refraction to decide on his lens implant power and type [2,3]. So, in the case of this patient, an outside-in approach was used where the scar and irregularity of the cornea were improved to make the cornea measurable followed by a lens implant into the aphakic eye to reach emmetropia (Figure 1) [2,4].

So, his aphakic eye was refracted on day one and week one post-op to a consistent measurement at +4.75, -1.50 x 165 to 20/30. We multiplied this spherical equivalent by a factor of 1.5 following the piggyback intraocular lens (IOL) formula to eventually go with full confidence with a Toric lens implant aiming straight for emmetropia [5,6].

According to IOL master, the power of the emmetropic lens predicted was +9.5D while A-Scan suggested the emmetropic power of +13D. We used the Piggyback IOL formula to calculate a SN6AT4 model with the power of +7.00, -2.25 x 059 which landed this patient at 20/20 without the need for any glasses or contact lenses [7]. The patient still had his keratoconus cornea, albeit stabilized with his previous Intacs procedure. And, our long-term goal was achieved permanently with the removal of his cataract and ICL to a stable and cherished endpoint.

## Discussion

In a patient like this, with multiple previous surgeries, many of which impacted the cornea adversely both in anatomy and measurability, the norm would be to offer a corneal transplant as the definitive treatment. Though a corneal transplant would provide some relief; its relative invasiveness along with potential comorbidities and lowered lens power predictability towards an accurate cataract surgery in this individual, that option was discussed as the last resort. In an extensive review with the patient, not only regarding the guarded prognosis, but also predictability, it was decided to intelligently manipulate the optics using the patient’s compromised anatomy in a staged goal of accurate emmetropic endpoint [2].

So, deciding that his eventual cataract surgery is our ultimate optical endpoint, we had to reach there with as much accuracy as we could using the patient’s current corneal and anterior segment status. In order to be predictable for the cataract lens implant, it was mandatory for us to make the cornea measurable. To do this, we embarked on a surface laser advanced surface ablation (ASA) technique following the manual refraction, to simultaneously sculpt the cornea while clearing the scar. This procedure itself improved the patient’s vision and perception to such an extent that he decided to delay his cataract surgery. We took advantage of this time period and measured him at different intervals to determine the stability and clarity of the cornea. 

One year later, he presented with a further decrease in vision due to his existent PSC. Though his cornea was measurable, given so many past corneal surgical failures, we did not want to take a chance in compromising my accuracy and hence decided to proceed with staged cataract surgery. During cataract surgery, first his previous ICL was explanted which on removing the cataract left him aphakic with an intact posterior capsular bag [3,4]. The sutureless corneal incision was further reinforced with ReSure sealent since the Intacs ring was very close to the incision. The patient was further refracted on day one and one-week post-op to determine the stability of refraction and using the secondary lens implant formula we determine a toric implant of SN6AT4 lens implant which was placed in the prepared capsular bag and aligned to the determined axis [5,6]. The endpoint of this process led the patient to see 20/20 without glasses and he was ecstatic at his outcome especially given the brief and staged techniques albeit in the right direction of the future anyways (Cataract surgery).

## Conclusions

This case is an example of how despite numerous previous failed surgeries a patient’s potential for vision can be pursued using carefully planned techniques without giving in to more invasive surgery, decreasing patient expectation, or the knee-jerk reaction of reversing all previous surgeries at the cost of vision.
